# Professional Fees and Fee Bills

**Published:** 1906-01

**Authors:** Robert H. M. Dawbarn

**Affiliations:** New York.


### PROFESSIONAL FEES AND FEE BILLS.

By ROBERT H. M. DAWBARN, M. D., New York.

In selecting a topic for discussion, and having been asked to make it one of mutual interest, it has occurred to me that possibly so prosaic a matter as the business end of our profession, or the doctor's obligation to himself, because almost wholly neglected in addresses, may be one not entirely unattractive. First of all, we are medical men and specialists. But in the second place, we are necessarily business men, with families to support, to educate and, later, to start in life; and the very practical questions, when to present our bills, and upon what principles of equity to fix the amounts to be charged therein, deserve attention. There are still gentlemen who feel that these matters are unworthy of us; that the "honorarium" (guineas handed in the sealed envelope), and also the legal disabilities formerly sadly handicapping those physicians who endeavored to compel collections against dishonest patients, constituted the proper condition of things, and that it is degrading to our profession to enforce our rights. But an increasing majority of physicians have abandoned the old-time view, and feel that to demand in all frankness what we have earned is no more unworthy of our dignity than it is derogatory to the dignity of the legal profession. The cases seem to me exactly parallel. Even to-day the doctor's bill is considered last of all. This is accounted for without difficulty. One is reminded of the deacon of accumulative instincts, who upon being reproached with his systematic neglect of the contribution plate, explained that the Lord did not press him so hard as did his other creditors!

As a very young practitioner I sent my bills four times a year. After a while I concluded to try the plan of rendering accounts, upon strict business principles, upon the first of every month, and of having engraved upon my billheads "Bills presented monthly," by way of indicating that nothing invidious is to be inferred by the patient in any instance. In no case has this plan induced death from heart failure at the shock of finding the physician to be also a man of business. On the contrary, I am satisfied, after many years of trial, that my patients—the honest ones, who are always a great majority —prefer this plan. Should an error in bookkeeping arise, it enables them to go back in memory over the brief interval since the preceding bill, and correct it. Moreover, it is unquestionably easier for them to pay and for the doctor to collect ten dollars twelve times a year than one hundred and twenty dollars once a year. And further, we should remember that gratitude, like alcohol, though a heart tonic, is volatile, and we should use it while it lasts.

By this plan, and with ordinary prudence, I do not have much above five per cent. of bad debts annually, which is a remarkably low percentage for a surgeon.

Most doctors and dentists are their own bookkeepers. As a class they do not earn their salary as such, and should discharge themselves. The tired, overworked practitioner will always postpone until that more convenient time which so seldom comes this particularly unpleasant duty. I believe that it will pay all, except those just beginning, and hence with very small practices, to hire an accountant by the year, turning over to him the pocket day book at the end of each month, and giving him a schedule of prices for certain work, as a general guide. There are hundreds of bookkeepers who will be glad, in return for a trifling sum earned outside of their business hours, to do this work. The bills thus prepared are to be examined in review by you, just before mailing, in the accountant's presence.

This naturally brings up the question of our fees, which needs agitation, if ever a topic did. Even the best brains of our profession are not paid as they deserve to be, and as, for example, eminent lawyers are. Take the instance of Senator Conkling. Being an honest man, he left the United States Senate in debt; but within the few years elapsing between then and his death in the great blizzard, he had, through legal fees, cleared himself, and left a fortune of more than one hundred thousand dollars. Such a thing would be an utter impossibility with us; and yet, in consideration of the good accomplished by our ablest men, and of the awful, the crushing responsibility of life or death sometimes upon their shoulders, no fee is high enough. It is ludicrous mental and moral obliquity to hold, as I have heard urged, that such work is beyond all price, and that this is why even their wealthy patients cannot attempt to compensate them for it.

I regard our greatest physicians as invariably underpaid. If by some simple suggestion, but born of genius wedded to experience, a brain is saved by the neurologist from insanity, a mind that otherwise must have entered oblivion, then a fee of one hundred thousand dollars, in the case of a millionaire, would be small indeed for that work. The specialist would in that case still be underpaid; would still have our commiseration—but not so much as usual.

In most such cases that brain specialist is, instead, lucky if he adds a twenty dollar fee to his slender bank account. And were he to charge a tithe, a mere vulgar fraction, of the first named sum, that patient, the man of wealth, would use his restored mentality in devising lurid invective to fit this peculiarly atrocious case of robbery!

On the other hand, an able consulting lawyer, who should win some transcontinental railway suit for this same millionaire, by raising some shrewd legal point, would name with calmness a fee of five figures, and it would be paid as a matter of course, and without unseemly wrath.

Who shall say that this picture is overdrawn? As to the rank and file of our profession, we exhibit our lack of business ability and invite the contempt of the ungodly in nothing so much as in our failure to combine for mutual protection against miserably insufficient prices for our work; as also in our lack of cordial mutual support. This remark applies equally to dentists and to all other specialists, as well as the general practitioner.

I have small patience when I hear, as I have repeatedly, the argument used that we should charge all people alike, without regard to their financial condition; that if we charge Mr. Smith fifty dollars for setting a broken bone, we have no right to charge Mr. Jones five hundred dollars for the same thing. Still less do I rejoice and give thanks when the speaker is a doctor (we all have met such) who maintains that a brother physician, in the case of a given wealthy patient whom he has cured, has "over-charged."

Let me illustrate by a parable. The Emperor William I. of Germany was fond, like Haroun al Raschid, of the "Arabian Nights," of mingling among his people unknown, or thinking himself so. Once upon a time, while wandering at night through a crowded market in Berlin, he priced the eggs offered by a certain old woman. Startled at the sum she named per dozen, he exclaimed, "Can it be that eggs are indeed so high as a mark apiece?" To which returned the handelsfrau, "Nay, your Majesty, eggs are plentiful enough. It is kings who are scarce."

Now, all can see the gross injustice herein attempted upon an inoffensive and harmless monarch. But the fable as I maintain applies to our case. We are not selling our brains over a counter at so much per think, or per dozen thinks. The Smith-Jones argument above mentioned comes with ill grace when directed against a profession which serves the poor without money and without price, to such an extent that one-fourth of the population of the great city of New York receive their medical attendance free!

Those who can pay should, therefore, as I believe, do so somewhat in proportion to the extent of their means; at least for operative and for consultation work involving unusual experience and ability.

This principle of a graded scale of charges is certainly the one approved of by the medical profession as a body, just as it is by the legal profession; the difference being that we have less of the courage of our convictions than have they. in carrying it out.

For example, most of us would not assess a poor clerk for expert work as much as we would charge a millionaire for that same attention. This fact being admitted, the principle just asserted is also admitted. And logically, if we charge that clerk (let us say) one cent, as a bill for our services, we should charge the man of money many thousands for the same thing, in order to maintain the same proportion.

This is not always feasible; but let none of us who admit that we do not have an identical fee bill for wealth and poverty alike, assail our more logical and consistent brothers who charge somewhat in real proportion to means as well as to the good accomplished. And let none of us do that shabby thing so common in our profession, of intimating that a certain fee is too high, and that we, if called, would have charged less!

One further point, often neglected; the family doctor or family dentist can better judge than the man whom he calls in consultation, as to the patient's fortune, and therefore, as to what would constitute a fair fee in a given case. It is a wise custom for the specialist to submit his proposed charge first to you who call him in, and to be guided by your advice in this matter. Should the operator offend the patient by the size of his bill, the doctor or dentist who has recommended him may suffer a vicarious punishment from the subject subsequently. Consequently the arrangement just named seems but courteous to the family medical attendant of whatever branch.

It has long seemed to me that much good would result if in all our medical schools one of the professors would address the graduating class, upon the eve of leaving their alma mater, giving a little lecture full of kindly advice upon just such practical topics as these now under discussion. It would assuredly help those young men to avoid certain financial blunders, for "just as the twig is bent the tree's inclined." It would also earn their subsequent and lasting gratitude, and by inducing sensible business methods, and concerted brotherly action for mutual protection in financial matters, would compel a respect in others for our calling as being one eminently worthy the best treatment, rather than, as now, the worst, at the hands of a business community.—*Items of Interest.*

